# Intraoperative checking of the first ray rotation and sesamoid position through sonographic assistance

**DOI:** 10.1007/s00402-022-04359-8

**Published:** 2022-03-11

**Authors:** Sergio Tejero, David González-Martín, Alfonso Martínez-Franco, Fernando Jiménez-Diaz, Gabriel Gijón-Nogueron, Mario Herrera-Pérez

**Affiliations:** 1 Head of Foot and Ankle Unit, Orthopedic Surgery and Traumatology Service, Hospital Universitario Virgen del Rocío, Av. Manuel Siurot, s/n, 41013 Sevilla, Spain; 2 School of Medicine, Universidad de Sevilla, Av. Sánchez Pizjuán, s/n, 41009 Sevilla, Spain; 3 Orthopedic Surgery and Traumatology Service, Hospital Universitario de Canarias, Carretera de la Cuesta s/n, 38320 Santa Cruz de Tenerife, Spain; 4 School of Medicine (Health Sciences), Universidad de La Laguna, Campus de Ofra, s/n, 38071 San Cristóbal de La Laguna, Spain; 5 Departamento de Enfermería y Podología, University of Seville, Sevilla, Spain; 6 Sport Sciences Faculty, Castilla La Mancha University, 45071 Toledo, Spain; 7 Departamento Enfermería y Podología, University of Málaga, Spain y IBIMA (Instituto de Invesigacion de Biomedicina de Malaga), Málaga, Spain; 8 Foot and Ankle Unit, Orthopedic Surgery and Traumatology Service, Hospital Universitario de Canarias, Carretera de la Cuesta s/n, 38320 Santa Cruz de Tenerife, Spain

**Keywords:** Hallux valgus, First metatarsal pronation, First tarsometatarsal fusion, Sonographic assistance

## Abstract

**Introduction:**

Hallux valgus (HV) deformity affects the orientation of the metatarsophalangeal (MTP) joint in three planes. Displacement in the coronal plane results in axial rotation of the first metatarsal, with progressive subluxation of the first MTP joint. Multiple techniques have been described to correct the malrotation itself. However, none of them have checked intraoperatively the final position of the first metatarsal head and sesamoids previous to the fixation of the Lapidus procedure or first metatarsal bone osteotomies. The aim of this article is to describe a novel technique to check the first ray rotation and sesamoids position through sonographic assistance.

**Materials and methods:**

Before fixation of the Lapidus procedure, with the ankle in maximal dorsiflexion, the surgeon takes the linear ultrasound probe and places it on the sole to visualize the sesamoids, which should be viewed at the same level, with the flexor hallucis longus (FHL) centered between both. Once the ideal position of the head of the first ray has been achieved, temporary fixation with K-wires is performed over the first TMT joint and M1–M2 joint for further sonographic verification of the sesamoids beneath the first metatarsal head. The height of the sesamoids relative to the second metatarsal head should be checked by sonographic control too.

**Results:**

Four patients were included. Three females and one male. Their mean age was 76.4 years (*R* 61–72). Their mean BMI was 29 (*R* 27.5–32.24). The mean IMA (intermetatarsal angle) was 18.2 (*R* 17.2–19) degrees and the mean MPA (metatarsophalangeal angle) was 50 (*R* 36–63) degrees.

**Conclusions:**

Sonographic assistance, is a widely available, inexpensive, and comparative imaging technique that can guide the first ray rotation and sesamoids position in HV surgery, theoretically improving radiological outcomes.

**Supplementary Information:**

The online version contains supplementary material available at 10.1007/s00402-022-04359-8.

## Introduction

Hallux valgus (HV) deformity affects the orientation of the metatarsophalangeal (MTP) joint in three planes [1]. The deformity in the coronal plane is the classical deformity that moves the great toe laterally with progressive subluxation of the first MTP joint [2]. In many cases, due to tarsometatarsal instability, the first ray could be rotated and dorsiflexed in addition to the main abduction deformity [3]. It creates increased load over tibial sesamoid and lesser metatarsal head with MTP plantar-plate injuries creating a painful and disabling condition that can negatively impact patients’ quality of life [4]. Surgical treatment has been found to significantly improve health-related quality of life

Recent studies using weight-bearing or simulated weight-bearing CT scans have better-demonstrated deformities in the sagittal and axial planes [7–9]. These studies have reported on differences in preoperative pronation of the first ray between normal and control patients [7, 9]. Algorithms for surgical treatment have historically been focused on the correction of the HV and intermetatarsal (IM) angles on two-dimensional radiographs [3, 10]. The modified Lapidus with innovative techniques such as Lapiplasty, [11] stabilizes the first ray at the tarsometatarsal joint, allowing the surgeon to correct rotation, sagittal plane, and coronal plane motion [11]. More recently, diaphysis or proximal osteotomies aim to control the pronation and dorsiflexion of the first ray [12]. Other surgeons prefer to have a preoperative weight-bearing CT scan, when possible, for planning the degrees needed to correct the rotation [7, 9]. However, up to date no surgical techniques have been designed a reliable method to check intraoperatively the final position of the first metatarsal head and sesamoids previous to the fixation of the Lapidus procedure or first metatarsal osteotomies [12].

This is the first work to describe a useful and simple method to control the position of the metatarsal head and sesamoids in the three-axis of space and avoid malposition during hallux valgus correction surgery through sonographic assistance.

## Methods

### Patient selection

The indications for the Lapidus procedure are severe instability of the first tarsometatarsal (TMT) joint affecting the medial column of the foot due to excessive movement of the first ray in the transverse (abduction > 20°), sagittal, and coronal (rotation > 10°) planes. In addition, it is indicated in symptomatic arthritis or conditions of the first TMT joint. In these cases, the sonographic assisted technique can be helpful to the surgeon to check the medial column and control the position of the metatarsal head before definitive fixation.

The use of this sonographic assistance would be contraindicated in situations of severe osteoarthritis or in grade 3 (sesamoid station) of the sesamoids, which can distort the image of their position, as well as their repositioning under the metatarsal head using a previous distal soft-tissue rebalancing procedure.

### Preoperative planning

The deformity of the first ray should be analyzed preoperatively using anteroposterior and lateral weight-bearing radiographs. If weight-bearing CT is available, it may be ideal to objectify the rotation of the first ray in loading as well as deformities in the coronal and sagittal planes.

### Operative and sonographic technique.

**Fig. 1:**
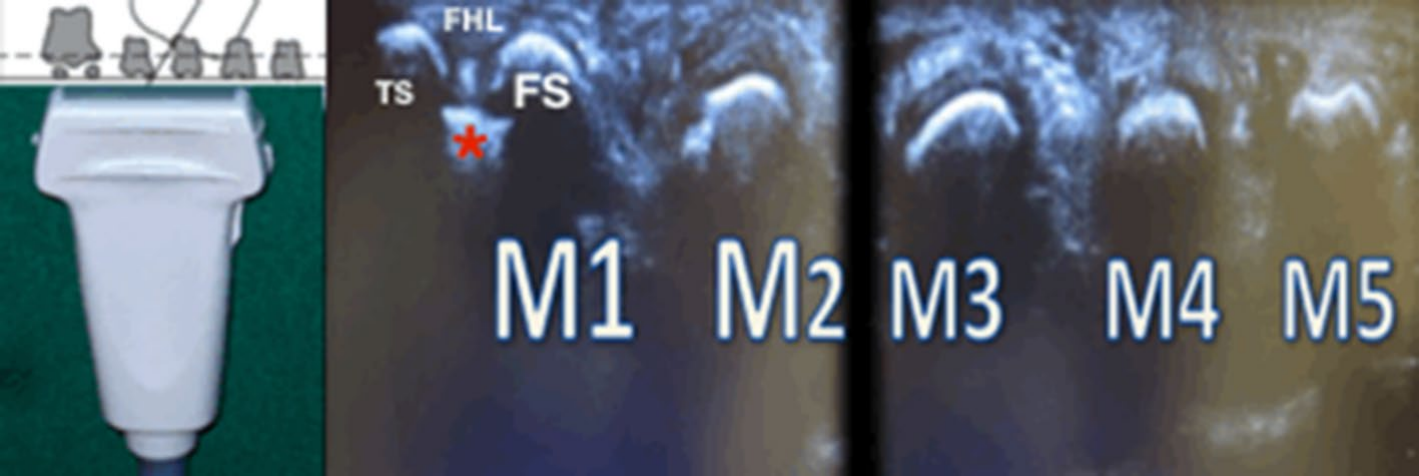
Scheme with ultrasonic probe showing the sesamoid bones and the first-fifth metatarsal heads (M1–M5). Note the tibial sesamoid (TS), fibular sesamoid (FS) centered on the crest of M1 (red asterisk) and the flexor hallucis longus (FHL) positioned between both sesamoid bones

**Fig. 2:**
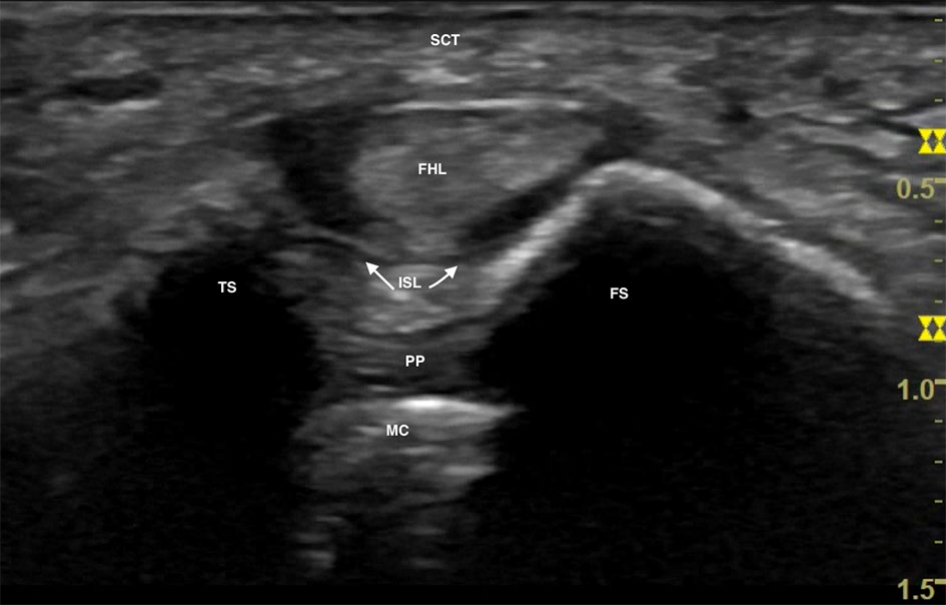
Sonographic view of the normal position of both sesamoids bones and the flexor hallucis longus (FHL) between tibial sesamoid (TS) and fibular sesamoid (FS). Note the metatarsal crest (MC) of the first metatarsal bone in line with the FHL. Intersesamoid ligament (ISL); plantar plate (PP)

Because the rotation of the first ray and sesamoids dislocation are two distinct entities within the same syndrome [13] they must be approached separately, so the first procedure is to reduce the sesamoids with the crest of the metatarsal head as a point of reference (Fig. 1). After the tourniquet ischemia in supine position, a lateral incision over the MTP joint is performed. The capsule should be opened by transecting the contracted lateral collateral ligament. The lateral metatarsal-sesamoid suspensory ligament must be identified and transected completely under direct vision. This procedure leaves the dorsal and plantar capsular attachment, the deep transverse metatarsal ligament, and the attachment of the adductor hallucis muscle completely intact. Second, after the bunionectomy, a medial capsular repair is accomplished using a U-shaped suture through of former medial metatarsal-sesamoid ligament for repositioning the sesamoid apparatus under the metatarsal head by fitting them on either side of the plantar crest of the metatarsal head (Fig. 2).

**Fig. 3:**
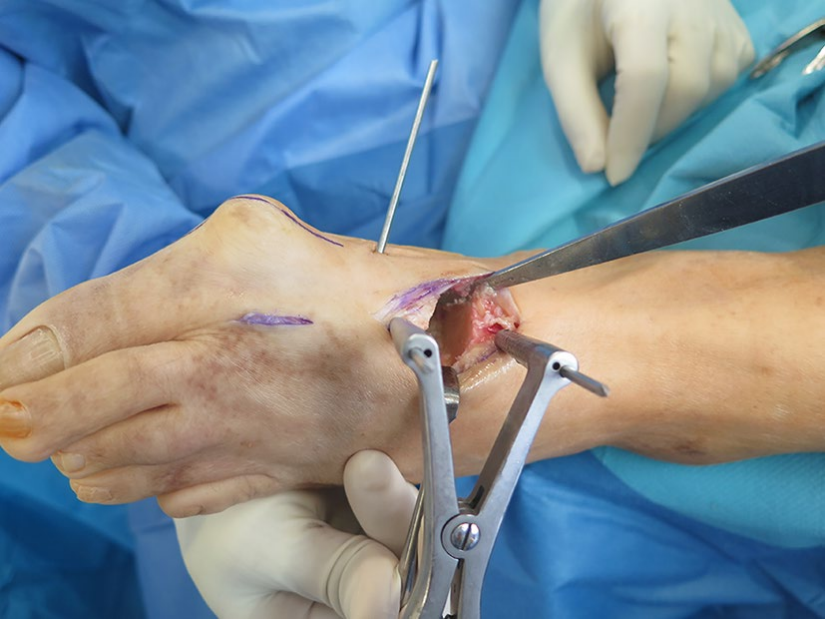
Parallel position of the dorsal K-wires with Hintermann spreader to let the debridement of the cartilage and the orthogonal cuts of the metatarsal base and the cuneiform bone. Note the perpendicular K-wire in the shaft of the first metatarsal bone used as a “Jokstick” for performing the supination, lateralization and plantar flexion of the head metatarsal

**Fig. 4:**
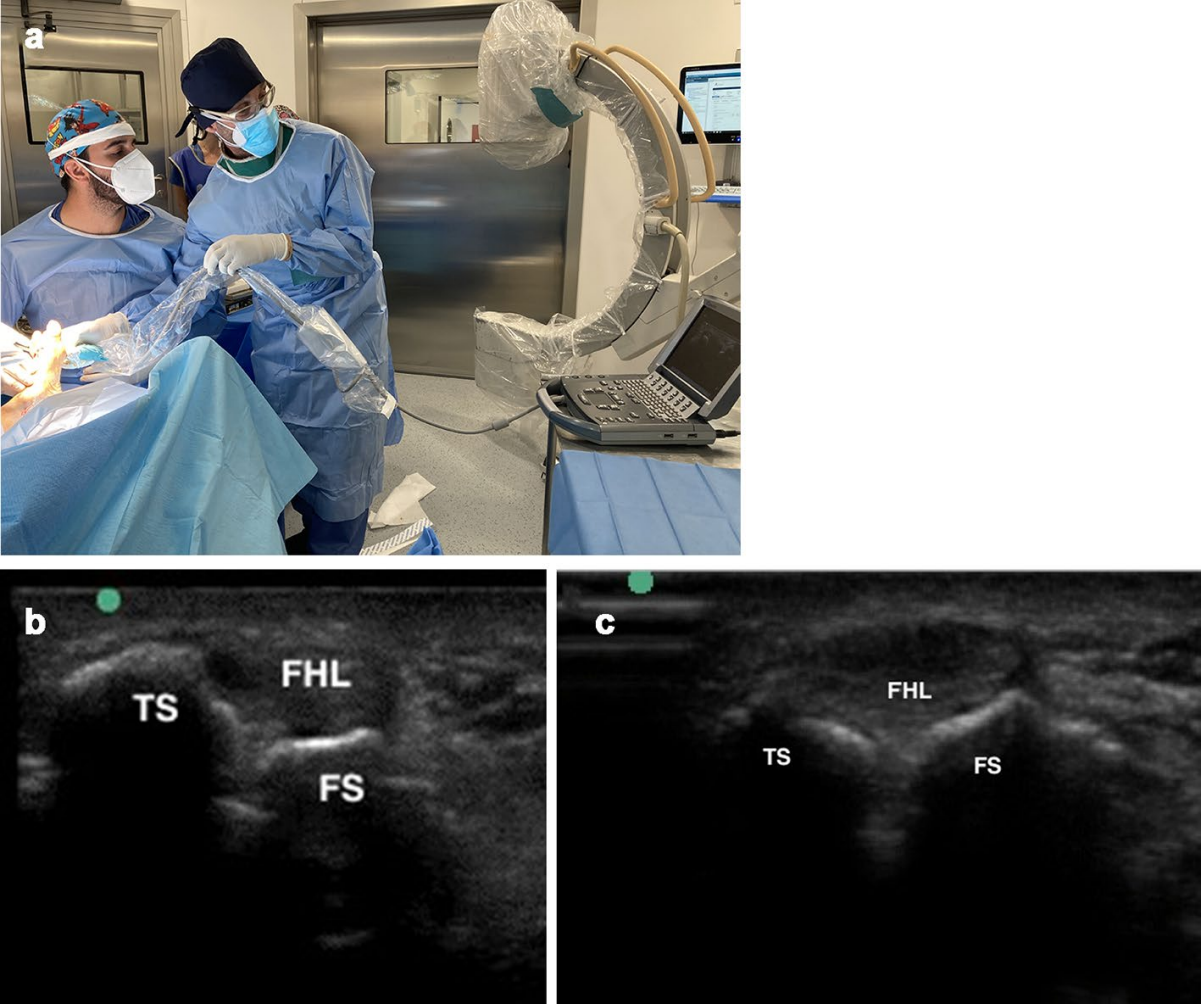
**a** An ultrasound view is carried out for checking the final position of the sesamoids and its level respect to lesser metatarsal heads. **b** Sonographic view of the sesamoid luxation in the context of hallux valgus. Note the tibial sesamoid (TS) and the fibular sesamoid (FS) positioned at different levels in axial plane and the FHL displaced laterally. **c** Sonographic view of both sesamoids bones and the flexor hallucis longus (FHL) between tibial sesamoid (TS) and fibular sesamoid (FS)

A dorsal or dorsomedial approach is then used to debride the joints of both first medial cuneiform (C1) and the base of the first metatarsal bone (M1). Before this, two 2.5 Kirschner wires (K-wires) are placed, parallelly and dorsally, on the axis of the first ray, one in the first cuneiform (C1) bone and the other in the metatarsal bone, which has a double function: (1) distraction using Hintermann retractor for resection of the plantar bone and (2) control of the rotation if it has also been planned previously with weight-bearing CT. A third K-wire is placed medially in the middle of the diaphysis of the first metatarsal which will be used as a Jokstick manner to lateralize the head (abduction control), supinate the metatarsal (rotation control), and plantar flexion (sagittal control) (Fig. 3, Video). At this point and with the ankle in maximal dorsiflexion, the surgeon takes the linear ultrasound probe and places it on the sole to visualize the sesamoids, which should be viewed at the same level, with the flexor hallucis longus (FHL) centered between both (Fig. 4). The height of the head of the second metatarsal can also be observed, which should be at the level of the sesamoids (sagittal control).

The frequency, depth, and focus of the ultrasound image should be adjusted. The focus should be adjusted at the level of the sesamoids. The linear ultrasound probe should be held firmly to simulate weight-bearing on the forefoot with the help of the hypothenar eminence, to achieve as much ankle dorsiflexion as possible. In turn, the probe should be held firmly, perpendicular to the first metatarsal, constantly sweeping from medial to lateral and from proximal to distal to check the position of both the sesamoids and the head of the second metatarsal. This will be the reference for the position of the first metatarsal in the sagittal plane. Not all structures can be seen at the same time in a single image, as is often the case with the ultrasound technique. Rotation is checked by observing the flexor hallucis longus between the two sesamoids and more deeply the hyperechoic ridge which shows the correct rotation of the metatarsal (Fig. 2).

**Fig. 5:**
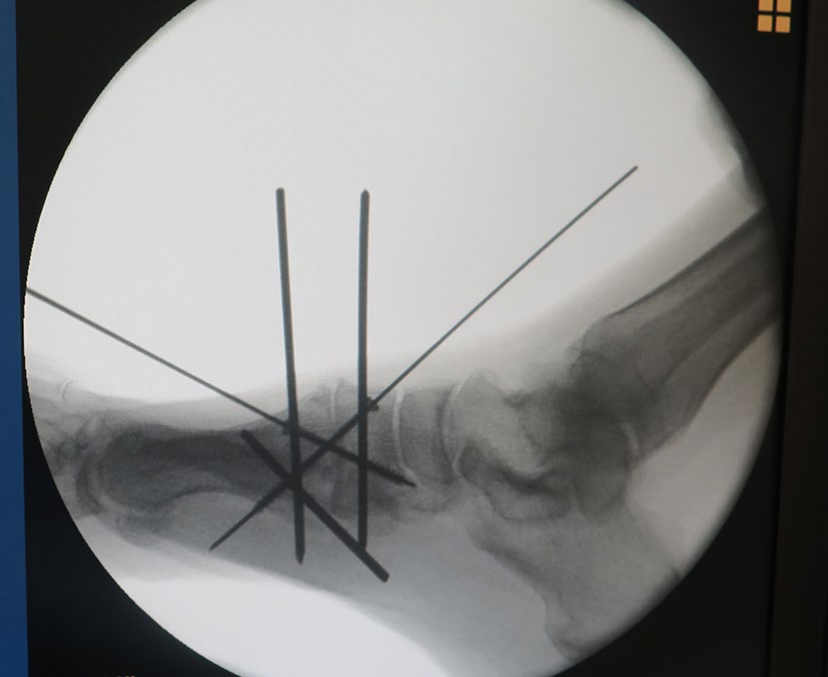
After the sonographic view of the correct position (Fig. 2a) of the sesamoid and FHL, one or two screws are placed through the tarsometatarsal (TMT) joint previous to the dorsomedial plate fixation

**Fig. 6:**
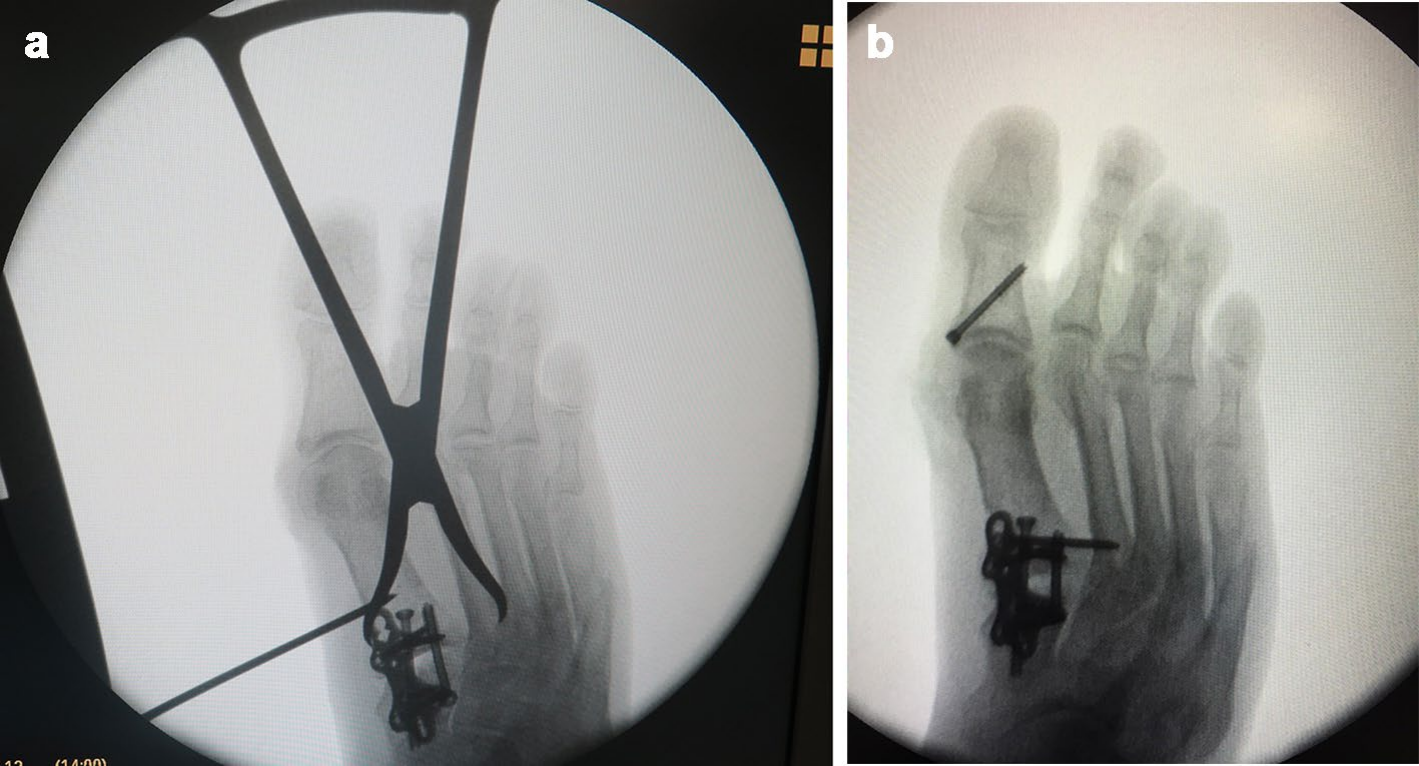
**a**–**b** If inter-cuneiform hypermovility is noted after TMT fixation, a screw between M1–M2 is placed

Once the ideal position of the head of the first ray in all three planes has been achieved, temporary fixation with K-wires is performed over the first TMT joint and M1–M2 joint for further sonographic verification of the sesamoids beneath the first metatarsal head. The height of the sesamoids relative to the second metatarsal head should be checked by sonographic control too. The intermetatarsal angle and the final position of the sesamoids under the metatarsal head should also be checked fluoroscopically. After this, a compression screw is placed between M1 and C1 (Fig. 5) and a dorsomedial neutralization plate completes the stable fixation. Finally, the first ray is abducted and if there is a gap > 4 mm between the medial and intermediate cuneiform (C1–C2), the cartilage is debrided at this level and M1–M2 is fixed to prevent recurrence (Fig. 6a, b).

The tourniquet ischemia is removed and, hemostasis and closure are performed.

### Postoperative imaging management

Patients operated on using this technique could start a progressive partial loading of 15 kg from the beginning, increasing weekly, until full loading is achieved at 6–8 weeks, at which time a control X-ray is requested. If a weight-bearing CT scan is available, it would be interesting to request this test at 3 months to check TMT consolidation and to verify the correct position of the sesamoids. Since few Foot and Ankle Units have this technology, ultrasound control of the position of the sesamoids can be performed at 3–6 months.

## Results

**Fig. 7:**
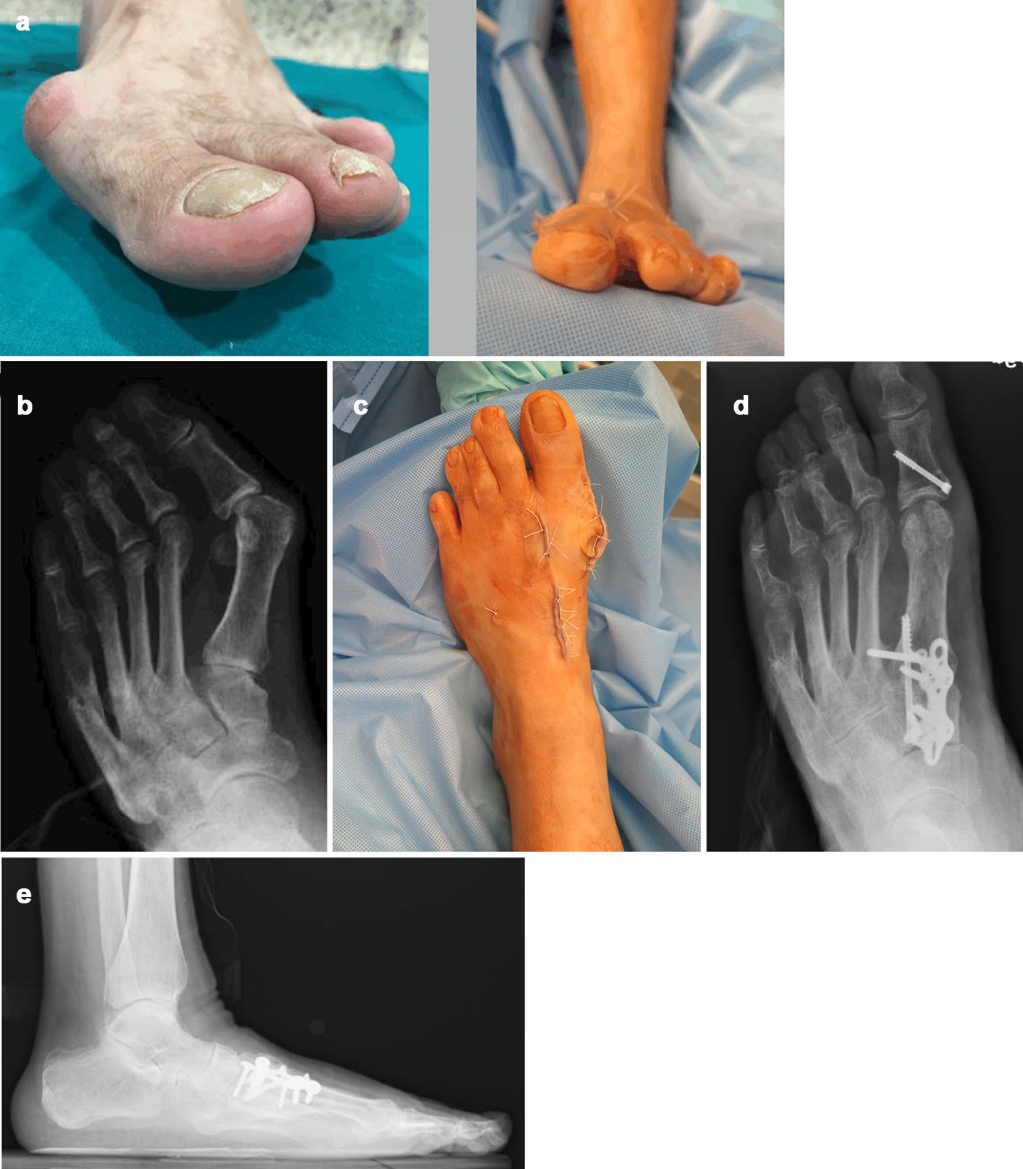
A 61-year-old male with severe TMT hypermovility and hallux valgus. **a** Note the preoperative pronation of the hallux and the postoperative correction; **b** Anteroposterior X-ray, note a consolidated stress fracture of the second and fifth metatarsal bones; **c** Clinical view after the operation; **d**, **e** Control X-rays at 12 months postoperatively

**Fig. 8:**
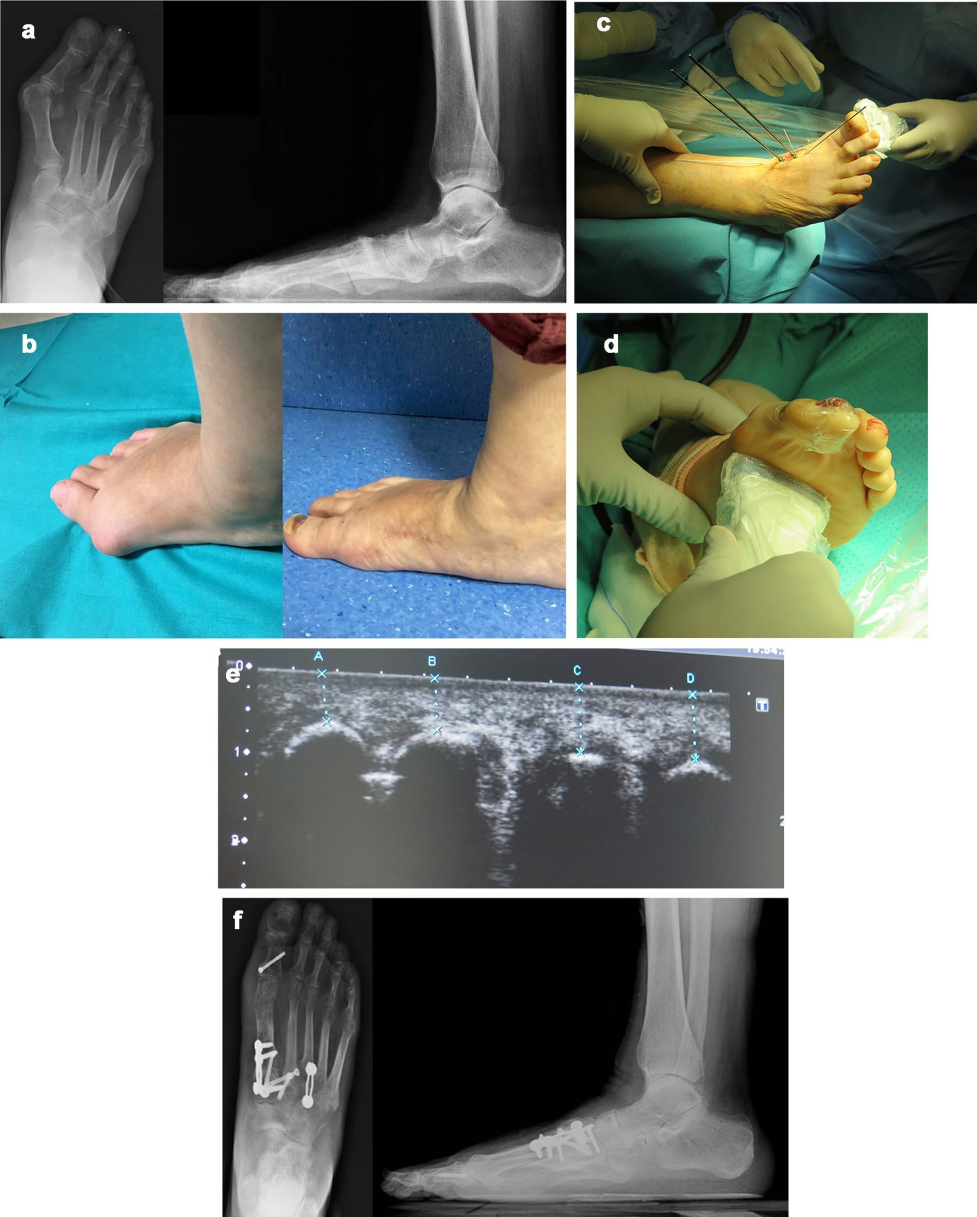
A 63-year-old female with hallux valgus. **a** Preoperative X-ray; **b** Clinical view before (left) and after (right) the operation; **c**, **e** Intraoperative checking of the first ray rotation and sesamoid position through sonographic assistance. The linear ultrasound probe should be held firmly to simulate weight-bearing on the forefoot with the help of the hypothenar eminence, to achieve as much ankle dorsiflexion as possible (**d**); **f** Control X-rays at 12 months postoperatively

Four patients were included (Table 1); three females and one male. Their mean age was 76.4 years (*R* 61–72); their mean BMI was 29 (*R* 27.5–32.24). The preoperative TMT status, sesamoid-metatarsal joint status, IMA and MPA can be found in Table 1. Clinical and radiological results of the case Nº 1 (Fig. 7a–e) and Nº2 (Fig. 8 a–f) at 12 months of the operation can be seen in the figures.

**Table 1:** Cases demographics, clinical, and radiological information

Case	Age	Weight (Kg)	BMI	Gender	Preop TMT status	Sesamoid-MTT status	IMA	MPA
1	61	91	32.24	Male	Instability plus OA	Mild OA	17.2	63
2	63	72	27.5	Female	Instability	NR	19	55
3	72	71	28.8	Female	Instability	NR	18	48
4	63	72	27.5	Female	Instability	NR	18.5	36

*BMI body mass index; *IMA* intermetatarsal angle; *MPA* metatarsophalangeal angle

## Discussion

This is the first technical surgical tip to describe a useful and easy method to control the position of the metatarsal head and sesamoids in the three-axis of space and avoid malposition of surgeries on the first ray.

Algorithms for surgical treatment have historically been focused on the correction of the HV and intermetatarsal (IM) angles on two-dimensional radiographs [3, 10]. The modified Lapidus with innovative techniques such as Lapiplasty, [11] stabilizes the first ray at the tarsometatarsal joint, allowing the surgeon to correct rotation, sagittal plane, and coronal plane motion [11]. More recently, diaphyseal or proximal osteotomies aim to control the pronation and dorsiflexion position of the first ray [12]. However, none of them have assessed intraoperatively the final position of the first metatarsal head and sesamoids previous to the fixation of the Lapidus procedure or first metatarsal bone osteotomies [12].

Recently, Conti et al. [13] described that the Lapidus procedure change pronation of the first ray in patients with HV but the reduction of the sesamoids to an anatomic position did not correlate with the amount of pronation of the first ray. Furthermore, they emphasized that although rotation deformity must be corrected an intraoperative method of exact correction is needed [13]. With the new technique described in this article, we propose a simple, practical, and effective way to control the first ray rotation using a resource available to the vast majority of orthopedic surgeons: the ultrasound.

Sonographic assistance is increasingly used in orthopedic surgery [14–16]. Thus, static, comparative, and dynamic information can now be obtained in many conditions. In addition, ultrasonography is a widely available, inexpensive, comparative, and dynamic imaging technique that involves no radiation exposure and has no other adverse effects. Therefore, we believe that we should optimize its use to improve surgical and functional outcomes in our patients.

The surgeon's concerns with the Lapidus technique are the consolidation of the joint, which has improved ostensibly with the new fixation systems using compression screws and blocked neutralization plates (dorsomedial or plantar plates), and the position in which the first metatarsal is fixed since its sequelae are disastrous. One of the advantages of this new technique that we propose, in addition to its main objective of controlling first metatarsal rotation, is that it does not increase the surgical time, does not greatly increase the cost of the operation, since it only requires a sterile arthroscopic sheath to cover the ultrasound probe and does not involve more harmful radiation for the patient.

On the other hand, the complications of this technique are no different from the Lapidus technique without sonographic assistance. Undercorrection associated with malalignment of the M1 in the sagittal or axial plane will lead to hallux valgus relapse and pain on the second MTP joint. Overcorrection in plantar position will cause a recalcitrant sesamoid pain. Because this technique is technically demanding, the authors consider it helpful to have intraoperative fluoroscopic and sonographic control to position the first metatarsal head oriented in all three planes of space.

However, it does require a learning curve for the orthopedic surgeon with ultrasound techniques. Possibly soon, ankle and foot surgeons will not only have an ultrasound scanner in the office but also in the operating theatre for procedures such as this, although it may also be useful in soft-tissue surgical procedures, or midfoot arthrodesis, as well as a powerful tool useful in surgical planning.

This new tool can be very helpful to the orthopaedic surgeon, however, it also has limitations that must be taken into account. First of all, it also requires the use of intraoperative X-rays, so that space in the operating theatre needs to be optimized as much as possible. Second, the learning curve, the correct patient selection, and the availability of sonographic assistance could be some of the obstacles to its generalization. On the other hand, the coronal rotation of the first metatarsal has been reported in the literature to be significantly different in the same foot between full weight-bearing status and non-weight-bearing status [17–19]. Thus, performing the ultrasound on the operating table is a limitation that although we have tried to solve by performing simulated weight-bearing with the linear ultrasound probe, it should be taken into account. On the other hand, in severe index minus, the linear probe is difficult to reach from the two sesamoids to the second metatarsal. Therefore, in these cases, a larger linear probe might be ideal.

## Conclusions

Sonographic assistance, is a widely available, inexpensive, and comparative imaging technique that can guide the first ray rotation and sesamoids position in HV surgery, theoretically improving radiological outcomes.

## Supplementary Information

Below is the link to the electronic supplementary material.

Supplementary file1 Video: Maneuver of the “Jokstick pin” for the correction of the pronation, abduction and plantar position of the first metatarsal head previous the fixation and sonographic checking. (MP4 59173 KB)
